# Cardiac Rehabilitation in the Modern Era: Optimizing Recovery and Reducing Recurrence

**DOI:** 10.7759/cureus.46006

**Published:** 2023-09-26

**Authors:** Amna Zaree, Shah Dev, Iqra Yaseen Khan, Mustafa Arain, Sohaib Rasool, Muhammad Asadullah Khalid Rana, Kainat Kanwal, Ridhi Bhagat, FNU Prachi, Piyush Puri, Giustino Varrassi, Satesh Kumar, Mahima Khatri, Tamam Mohamad

**Affiliations:** 1 Medicine, Shalamar Medical and Dental College, Lahore, PAK; 2 Internal Medicine, Jinnah Sindh Medical University, Karachi, PAK; 3 Medicine, King Edward Medical University (KEMU), Lahore, PAK; 4 Internal Medicine, Civil Hospital Karachi, Karachi, PAK; 5 Medicine, Bakhtawar Amin Medical and Dental College, Multan, PAK; 6 Medicine, Aziz Fatimah Medical & Dental College, Faisalabad, PAK; 7 Medicine and Surgery, Khawaja Muhammad Safdar Medical College, Sialkot, PAK; 8 Internal Medicine, Teerthanker Mahaveer Medical College and Reseach Center, Moradabad, IND; 9 Medicine, Guru Teg Bahadur Hospital, Delhi, IND; 10 Internal Medicine, Adesh Institute of Medical Science and Research, Bathinda, IND; 11 Pain Medicine, Paolo Procacci Foundation, Rome, ITA; 12 Medicine and Surgery, Shaheed Mohtarma Benazir Bhutto Medical College, Karachi, PAK; 13 Medicine and Surgery, Dow University of Health Sciences, Karachi, PAK; 14 Cardiovascular Medicine, Wayne State University, Detroit, USA

**Keywords:** rehabilitation, optimizing, recurrence, cardiovascular, recovery, review

## Abstract

Cardiovascular diseases (CVDs) continue to be a prominent issue in global health, emphasizing the necessity for efficient cardiac rehabilitation programs. This narrative review provides a detailed analysis of the current state of cardiac rehabilitation, focusing on maximizing recovery and minimizing the likelihood of recurrence. This paper examines the historical progression of cardiac rehabilitation, explores the epidemiological background of cardiovascular disease (CVD), and provides an overview of the many stages of the rehabilitation process. The assessment of patients plays a crucial role in healthcare, serving as a fundamental basis that incorporates medical, psychological, and social aspects. The utilization of risk stratification techniques further enhances this process. The present study investigates exercise training, particularly emphasizing the current recommendations and the mutually beneficial effects of aerobic and resistance regimens. In addition to physical therapies, this study emphasizes the importance of nutrition, lifestyle adjustments, and the significant effects of medication. Psychosocial assistance is a crucial element that addresses the significant psychological effects of cardiac disease and provides comprehensive techniques for overall well-being. Technological advancements are significantly transforming the domain of cardiac rehabilitation, encompassing the integration of wearable technologies and telemedicine solutions. The convergence of artificial intelligence and data analytics can enhance the customization of healthcare services. Through a comprehensive rehabilitation program, patients can achieve an improved quality of life and enhanced functional outcomes. However, it is essential to acknowledge that obstacles still hinder individuals from accessing and completing educational programs. Therefore, it is crucial to engage in a discourse on potential tactics that may be employed to address these issues, considering the various cultural and socioeconomic aspects that contribute to them. The analysis focuses on the economic dimension, examining the cost-effectiveness of rehabilitation programs and their congruence with healthcare policies. In anticipation of future developments, the study provides valuable perspectives on the prospective trajectory of cardiac rehabilitation. It delves into nascent patterns and examines the potential ramifications of precision medicine and genetics for tailoring treatment strategies to individual patients. In brief, this narrative review comprehensively examines the various dimensions of contemporary cardiac rehabilitation. It offers a comprehensive perspective on its significance in enhancing recuperation and mitigating the likelihood of the recurrence of cardiovascular ailments. The significance of this review lies in its ability to enhance patient outcomes, thereby making a valuable contribution to the worldwide endeavor to address the burden of cardiovascular disease.

## Introduction and background

The importance of cardiac rehabilitation has experienced significant growth within the contemporary healthcare scene. Cardiovascular diseases (CVDs) persistently impose a significant burden on public health, encompassing consequences that transcend beyond the physical symptoms individuals encounter [1]. Effectively managing these illnesses necessitates a comprehensive approach involving short-term recuperation and long-term prevention. Cardiac rehabilitation is a fundamental component of this framework, providing a comprehensive approach to enhance the recovery of people who have experienced cardiac events while simultaneously reducing the likelihood of future occurrences [2]. This narrative review aims to comprehensively explore the topic of cardiac rehabilitation in the present era. The review will focus on the historical evolution of cardiac rehabilitation, the epidemiological backdrop surrounding cardiovascular diseases (CVDs), and the various components that make up rehabilitation programs. This study emphasizes cardiac rehabilitation's growing importance in contemporary healthcare. Furthermore, this study aims to emphasize the most recent advancements, ongoing obstacles, and potential future direction of this subject, ultimately contributing to a comprehensive comprehension of how cardiac rehabilitation matches the overarching goals of modern healthcare.

In contemporary healthcare, cardiac rehabilitation has transitioned from being a supplementary element to a crucial cornerstone in the comprehensive care provided to those impacted by cardiovascular ailments [3]. These illnesses comprise a wide array of disorders, such as coronary artery disease, heart failure, and arrhythmias, and, combined, constitute the primary cause of death globally. In addition to directly impacting patients' well-being, cardiovascular diseases (CVDs) impose a significant financial burden on healthcare systems, society, and individuals. With the evolution of healthcare paradigms, there is a growing emphasis on preventative measures, patient-centered treatment, and the careful allocation of resources [4]. In this context, cardiac rehabilitation has emerged as a crucial technique that effectively matches these objectives. The notion of cardiac rehabilitation signifies a significant paradigm shift in the management of cardiovascular diseases [5]. The program goes beyond the boundaries of conventional medical intervention by providing a complete approach that caters to the multifaceted requirements of those who have undergone cardiac events. This treatment involves a comprehensive approach to recovery, incorporating not just the physical components but also addressing psychological well-being, social support, lifestyle alterations, and risk factor management [6]. Therefore, cardiac rehabilitation is the intermediary that connects the gap between the initial stage of medical intervention and the ongoing process of treating and alleviating heart disease [7].

The significance of cardiac rehabilitation becomes progressively apparent within this particular framework. The statement above signifies a shift towards prioritizing prevention, empowerment, and comprehensive treatment [8]. In the context of healthcare systems contending with the increasing prevalence of chronic diseases, it is crucial to comprehend the intricate aspects of cardiac rehabilitation [9]. This narrative review aims to offer a thorough and up-to-date examination of cardiac rehabilitation within the current dynamic environment and elucidate its significance in enhancing recuperation and mitigating the likelihood of recurrence in individuals affected by cardiovascular ailments.

## Review

Historical perspective: the evolution of cardiac rehabilitation

The historical perspective surrounding cardiac rehabilitation is characterized by a notable change process, characterized by several advancements and improvements over several decades, in direct response to the increasingly significant impact of cardiovascular illnesses (CVDs) [1]. The discipline of cardiac rehabilitation has seen significant transformation, progressing from its humble origins in the early 1900s to the current state of advanced, interdisciplinary programs. This historical analysis aims to shed light on the significant milestones, advancements, and individuals who have played a crucial role in shaping the field of cardiac rehabilitation from its early beginnings to its present position as an essential element of contemporary cardiovascular healthcare [2].

Early Roots and Pioneers

The origins of cardiac rehabilitation can be attributed to the groundbreaking contributions of several healthcare practitioners during the early 1900s. Dr. Jeremy Morris, an esteemed epidemiologist hailing from the United Kingdom, made a significant contribution to the recognition of the influence of physical activity on cardiovascular well-being [3]. During the latter part of the 1940s, Morris undertook a seminal investigation wherein he compared the health outcomes of London bus conductors, who engaged in physical activity as part of their occupation, with those of bus drivers, who did not [4]. The study revealed a notable reduction in the prevalence of heart disease among the conductors. This pioneering study established the fundamental principles that physical activity and exercise have the potential to be utilized in the treatment and control of cardiovascular disorders [5].

Post-World War II Era: Emergence of Cardiac Rehabilitation Programs

During the period following World War II, he witnessed the establishment of structured cardiac rehabilitation programs. Dr. Paul Dudley White, a distinguished cardiologist, saw the prospective benefits of cardiac rehabilitation for patients in the United States [1-3]. Consequently, he took the initiative to establish the inaugural cardiac rehabilitation program at Massachusetts General Hospital in Boston during the latter part of the 1940s. White's objective was to establish a methodical and closely monitored physical activity regimen to aid the rehabilitation process of those who have experienced cardiac events [1,5]. This groundbreaking initiative catalyzed the creation of other initiatives throughout the United States.

1960s-1970s: Expanding Horizons

During the 1960s and 1970s, there was a notable increase in the establishment of cardiac rehabilitation programs, both domestically in the United States and on a global scale [6]. The provision of cardiac rehabilitation expanded beyond exclusive academic medical institutes, making it more readily available to a broader range of patients. Exercise stress testing has played a pivotal role in enhancing the precision of evaluating patients' cardiovascular fitness and facilitating the development of customized exercise prescriptions [2-6]. During this era, there was a notable increase in the integration of cardiac rehabilitation programs within outpatient facilities, extending their scope and influence.

1980s-1990s: Guidelines and Standardization

The decades of the 1980s and 1990s were characterized by a significant shift in the field of cardiac rehabilitation, as evidenced by the release of influential guidelines and the establishment of standardized care protocols [7]. In 1986, the American Association of Cardiovascular and Pulmonary Rehabilitation (AACVPR) released its inaugural recommendations, which established a comprehensive structure for the development of programs and the care of patients. The guidelines underscored the significance of including multidisciplinary teams comprising exercise physiologists, nurses, dietitians, and psychologists [8]. During a similar period, the endorsement of cardiac rehabilitation in secondary prevention by the American Heart Association (AHA) and the American College of Cardiology (ACC) further established its position as a widely accepted intervention [9].

21st Century: Advancements in Technology and Multidisciplinary Care

The onset of the 21st century marked the commencement of a distinct epoch in the field of cardiac rehabilitation, marked by notable progressions in technological innovations and an emphasis on comprehensive, interdisciplinary approaches to patient treatment [10]. The utilization of wearable devices and telehealth solutions has become more significant in monitoring patients' progress and promoting adherence to exercise programs. These advancements increased the convenience of rehabilitation and strengthened the capacity to monitor patient results [11]. Furthermore, incorporating artificial intelligence and data analytics has presented potential avenues for tailoring exercise prescriptions and enhancing therapy strategies.

Global Impact and Recognition

The acknowledgment of cardiac rehabilitation as a vital element of cardiovascular healthcare transcended national borders, garnering momentum on an international level [11]. Different nations have formulated guidelines and accreditation criteria, modifying cardiac rehabilitation programs to align with their healthcare systems and cultural situations. The European Association of Preventive Cardiology (EAPC) has played a leading role in advocating for advancing cardiac rehabilitation in Europe [12]. In the meantime, efforts such as the Global Cardiac Rehabilitation Registry (GCCR) have been undertaken to establish uniformity in data gathering and promote global cooperation in research and program advancement.

Contemporary Cardiac Rehabilitation: A Multifaceted Approach

In the present day, contemporary cardiac rehabilitation is distinguished by its comprehensive approach to cardiovascular healthcare. It incorporates various components, including structured exercise programs, detailed evaluations, risk factor management, psychosocial support, and lifestyle counseling [13]. These comprehensive programs benefit those suffering from cardiovascular ailments such as coronary artery disease, heart failure, and heart transplantation [14]. The contemporary approach to cardiac rehabilitation places significant importance on developing individualized care plans specifically designed to address each patient's distinct requirements and objectives [15]. This emphasis serves to highlight the patient-centric nature of modern cardiac rehabilitation practices.

Impact and Challenges Ahead

Extensive documentation exists about the influence of cardiac rehabilitation on patient outcomes. Numerous studies consistently indicate that cardiac rehabilitation programs yield notable enhancements in individuals' quality of life, substantial reductions in mortality rates, and notable decreases in hospital readmission rates [12]. Notwithstanding the evident advantages, several obstacles continue to endure. An inequitable distribution of access to cardiac rehabilitation programs exists, characterized by inequities associated with geographical location, socioeconomic level, and healthcare infrastructure [13]. Promisingly, continuing endeavors aim to mitigate these discrepancies and enhance the accessibility of services to marginalized people.

Future Directions: Precision Medicine and Beyond

With the ongoing evolution of the healthcare profession, cardiac rehabilitation is positioned to embrace forthcoming developments. The advent of precision medicine and genomics has prospects for customizing rehabilitation protocols according to an individual's genetic and molecular characteristics [14]. This has the potential to augment the customization and efficacy of cardiac rehabilitation. Furthermore, the continuous investigation of innovative interventions, such as regenerative therapies and improved pharmacological agents, can broaden the range of rehabilitation by including emerging treatments for cardiovascular disorders [15].

To conclude, the progression of cardiac rehabilitation from its initial origins to the contemporary era serves as a testament to the efficacy of inventive practices, extensive research, and a comprehensive approach to patient well-being. The initial idea originated from recognizing the advantages of physical activity and has evolved into a multidisciplinary domain encompassing several aspects of cardiovascular health, including the physical, psychological, and social components [16]. Cardiac rehabilitation, boasting a substantial chronicle of accomplishments, persists as a vital constituent of contemporary healthcare while harboring the potential for further advancements in tailored and efficacious interventions [17]. The entity mentioned above symbolizes optimism for persons afflicted with cardiovascular ailments, presenting the possibility of an improved state of well-being and a more gratifying existence.

Epidemiology of cardiovascular disease

Cardiovascular diseases (CVDs) pose a substantial global health burden, exerting a profound influence on individuals, communities, and healthcare systems around the globe. To have a comprehensive understanding of the current importance of cardiac rehabilitation within the framework of contemporary healthcare, it is imperative to examine the epidemiology of cardiovascular disorders [18]. This section analyzes the current global prevalence and effect of cardiovascular diseases (CVDs) globally and within specific areas. Furthermore, this study delves into the socioeconomic implications of heart disease on societies, emphasizing the pressing need for efficient solutions in prevention and management [19].

Global Prevalence and Impact

Cardiovascular illnesses have established themselves as the predominant cause of mortality globally. According to the World Health Organization (WHO), the latest available data reveals that cardiovascular diseases (CVDs) constitute around 31% of the total global mortality rate [3-5]. The statistical data indicates that over 18 million individuals succumb to heart disorders yearly, encompassing coronary artery disease, heart failure, and stroke. Furthermore, it is essential to note that the impact of cardiovascular diseases (CVDs) extends beyond high-income countries, as people in low- and middle-income nations are disproportionately affected by this burden [6]. The abovementioned figures highlight the extensive and all-encompassing prevalence of cardiovascular diseases (CVDs) as a matter of worldwide significance in public health. In addition, cardiovascular diseases (CVDs) impose a significant financial burden. According to the World Economic Forum, if current trends continue, the worldwide economic burden of cardiovascular diseases (CVDs) will surpass one trillion dollars annually by 2030 [7]. The financial burden comprises various components, including healthcare expenses, losses in productivity, and the indirect costs associated with early mortality. Heart illnesses have a profound impact, affecting individuals and societal structures extensively [8].

Regional Disparities

Cardiovascular diseases (CVDs) pose a significant worldwide health issue. Yet, the distribution of this burden is not uniform among different countries and populations. Disparities in the prevalence and consequences of cardiovascular disease (CVD) can be attributed to variations in risk factors, healthcare infrastructure, and the availability of preventive and therapeutic therapies [1-5]. There is a notable disparity in age-adjusted death rates from cardiovascular diseases (CVDs) between high-income and low- and middle-income nations. Nevertheless, discrepancies may continue within nations, influenced by socioeconomic variables, geographical location, and ethnic background. Significant advancements have been achieved in reducing cardiovascular disease (CVD)-related mortality in North America and Western Europe during recent decades [3-7]. The good trends observed can be attributed to various factors, including advancements in healthcare delivery, managing risk factors, and developing superior treatments. On the other hand, it is worth noting that sub-Saharan Africa and South Asia are still grappling with an upward trend in cardiovascular diseases (CVDs) [8]. This can be attributed to various factors, including urbanization, alterations in dietary patterns, and the growing prevalence of risk factors such as hypertension, diabetes, and cigarette consumption.

Socioeconomic Burden

The socioeconomic impact of heart disease extends beyond the immediate financial implications associated with medical treatment. The phenomenon under consideration involves a multifaceted interaction of economic, social, and human factors, which can dramatically impact people, families, and broader society [9]. Healthcare expenditures associated with managing cardiovascular diseases, encompassing hospitalization, medication, surgical interventions, and rehabilitation, substantially burden healthcare budgets. The demand for specialized cardiac treatment, which frequently entails expensive interventions like angioplasty or coronary artery bypass grafting, significantly contributes to healthcare costs. Cardiovascular disorders have been found to result in untimely impairment and mortality, thereby depriving society of the prospective contributions that individuals affected by these conditions could have made to the labor force and the economy [10]. The economic impact is further exacerbated by the loss of production resulting from absenteeism and reduced job performance. The impact of heart disorders on people's quality of life can be substantial. The presence of functional limits, the requirement for continuous medical care, and the psychological consequences associated with the experience of living with a chronic condition can reduce overall well-being and life satisfaction. Caring for patients with cardiovascular diseases (CVDs) frequently rests on family members and caregivers, resulting in a significant burden [11]. This phenomenon can lead to psychological distress, economic burden, and disturbances in daily functioning, impacting the individual undergoing the experience and their social network.

Socioeconomic determinants, such as income, education, and healthcare accessibility, significantly influence the distribution of heart disease risk and its disproportionate burden on specific populations [12]. Vulnerable populations, characterized by limited access to resources and residing in underserved locations, frequently encounter elevated incidences of heart disease and experience suboptimal health outcomes. The coexistence of cardiovascular illnesses with other chronic ailments, such as diabetes and obesity, is common [13]. The intricate nature of this phenomenon can lead to an exacerbation of health-related difficulties and expenses. Effectively addressing the socioeconomic burden associated with heart disease necessitates implementing a comprehensive approach that transcends the boundaries of clinical care. Implementing initiatives focused on primary prevention, early identification, patient education, and targeted therapies for high-risk populations is imperative [14]. Efforts of this nature should consider the broader determinants of health, encompassing social and economic issues, which have a role in the emergence and worsening of cardiovascular diseases.

Epidemiology sheds light on the prevailing reality surrounding cardiovascular diseases, indicating that heart disorders remain a prominent worldwide health concern, affecting many individuals and imposing considerable economic implications [14]. Regional and population-specific variations exist in the occurrence and effect of cardiovascular diseases (CVDs), which can be attributed to various influencing variables. To effectively tackle this significant health issue, embracing a holistic and all-encompassing strategy that involves preventative measures, timely intervention, and equal availability of high-quality healthcare services is crucial [15]. Cardiac rehabilitation is an essential element of contemporary healthcare, serving a crucial function in moderating the effects of cardiovascular diseases (CVDs), enhancing patient outcomes, and reducing the socioeconomic consequences of heart disease for individuals and societies [16].

Phases of cardiac rehabilitation

Cardiac rehabilitation is a comprehensive and diverse procedure in various stages, each with goals and interventions customized to the patient's condition and development. These phases encompass the entire spectrum of healthcare, from the immediate aftermath of a cardiac event to the long-term management of cardiovascular well-being [16]. This section delves into the many stages of cardiac rehabilitation, specifically the inpatient, outpatient, and maintenance phases. In this discussion, we aim to clarify the specific goals of each phase and explore the significant contribution made by multidisciplinary teams in providing complete care throughout the rehabilitation process [17].

Inpatient Phase

The inpatient phase is when a patient is admitted to a healthcare facility for intensive medical care and treatment. The commencement of the inpatient phase of cardiac rehabilitation occurs promptly following a cardiac event, such as a myocardial infarction, cardiac surgery, or the identification of a notable cardiac ailment [18]. The main goals of this initiative encompass stabilization, identification of risk factors, and early education. Upon admission to the hospital, patients are promptly provided with medical care, which frequently encompasses operations such as angioplasty or coronary artery bypass grafting [19]. Concurrently, healthcare practitioners commence the evaluation of cardiovascular risk factors, including hypertension, hyperlipidemia, and diabetes, while offering instruction on lifestyle adjustments and medication administration. During the inpatient period, a collaborative effort is undertaken by a team of healthcare providers from several disciplines [20]. The composition of this team often encompasses professionals such as cardiologists, nurses, nutritionists, physical therapists, and social workers. Cardiologists are responsible for the supervision of medical management. At the same time, nurses are tasked with monitoring vital signs and administering drugs [21]. Dietitians assess individuals' dietary patterns and recommend promoting cardiovascular health through nutrition. Physical therapists evaluate patients' mobility and provide suitable exercises as deemed necessary. Social workers and psychologists provide individuals with emotional assistance and address psychosocial elements that could influence the healing process [22].

Outpatient Phase

The outpatient phase refers to the stage of medical treatment where patients get care and treatment without being admitted to a hospital or healthcare facility. After completing the inpatient phase, individuals proceed to the outpatient phase of cardiac rehabilitation. The abovementioned period usually commences shortly after the patient's release from the medical facility [23]. It may persist for several weeks or months. This intervention's objectives include addressing physical and psychosocial recovery, modifying risk factors, and implementing an organized exercise training program. During the outpatient phase, the composition of the multidisciplinary team is augmented to incorporate supplementary specialists [24]. Exercise physiologists are essential in developing and overseeing exercise programs customized to meet individual patients' unique requirements and physical capabilities. Healthcare professionals oversee the cardiovascular reactions of patients during physical activity and ensure the implementation of a safe and gradual exercise program [25]. Nurses persist in delivering medical oversight, encompassing the administration of medications and the surveillance of essential physiological indicators throughout exercise sessions.

Dietitians persist in providing nutritional advice and attending to individuals' specific dietary requirements and preferences. Psychologists and counselors are accessible resources that offer psychosocial assistance, specifically targeting emotional concerns such as anxiety and depression, which frequently manifest following cardiac incidents [26]. Additionally, they aid in facilitating stress management techniques and coping skills. During the outpatient phase, patients are instructed on heart-healthy habits, encompassing nutrition, smoking cessation, and medication adherence. Furthermore, the diligent monitoring and management of risk factors, such as hypertension, hyperlipidemia, and diabetes, are frequently undertaken, generally in collaboration with primary healthcare practitioners [27]. The objective is to enhance patients' capacity by equipping them with the necessary knowledge and abilities to embrace a lifestyle promoting cardiovascular health, diminishing the likelihood of experiencing subsequent cardiovascular incidents.

Maintenance Phase

In the maintenance phase, ongoing efforts are made to sustain or preserve the current state or condition. The maintenance phase encompasses the extended duration of cardiac rehabilitation, surpassing the confines of the structured outpatient program [28]. The key aims of this program are to maintain the progress made in previous stages, encourage continual adjustments to one's lifestyle, and facilitate the incorporation of heart-healthy habits into everyday routines [29]. During the maintenance phase, patients are advised to maintain a consistent exercise routine, adhere to dietary guidelines, and effectively control their cardiovascular risk factors. Exercise programs have the potential to shift from supervised sessions to autonomous, home-based routines, albeit with the understanding that the continued availability of exercise facilities and guidance from healthcare professionals are still considered significant assets [30].

Despite an increased focus on regular follow-up and monitoring, multidisciplinary teams remain involved in the maintenance phase [31]. Cardiologists and primary care doctors oversee ongoing medical management and drug adjustments. Dietitians can offer regular nutritional counseling sessions to strengthen dietary habits. Psychologists and counselors are accessible resources for attending to individuals' psychological and emotional well-being, particularly those experiencing anxiety or depression about their cardiovascular health [32]. The crucial aspect of the maintenance phase involves formulating an individualized and enduring self-care strategy. It is strongly advised that patients regularly monitor their health, exhibit awareness of indicators that may signal the presence of cardiovascular ailments, and promptly seek medical intervention when deemed required [33]. In addition, individuals are provided with continuous educational resources and assistance in effectively managing stress, sustaining a healthy body weight, and making well-informed decisions regarding lifestyle preferences.

Additionally, the maintenance phase acknowledges the significance of continuous social support. Patients can participate in support groups or community-based initiatives promoting peer interactions and exchanging shared experiences. These relationships can catalyze inspiration, support, and responsibility as individuals endeavor to sustain their cardiovascular health-promoting behaviors in the long run [34]. Cardiac rehabilitation encompasses various stages, each serving unique purposes and significantly contributing to the overall recuperation and welfare of those afflicted with cardiovascular ailments. The initial phase of inpatient care centers on achieving stability and providing fundamental education. The subsequent outpatient phase places emphasis on structured exercise training and the modification of risk factors. Lastly, the maintenance phase aims to support the long-term sustainability of behaviors that promote a healthy heart [35]. Multidisciplinary teams, consisting of diverse healthcare professionals, assume essential roles across the spectrum of treatment. The collaboration between healthcare professionals guarantees that patients receive complete and individualized assistance, encompassing cardiovascular health's physical, psychological, and social aspects throughout every phase of their rehabilitation process [36]. By strategically aligning with these aims and harnessing the specialized knowledge of multidisciplinary teams, cardiac rehabilitation emerges as a crucial element of contemporary healthcare, facilitating optimal recovery and mitigating the likelihood of recurring cardiovascular incidents [37].

Patient assessment in cardiac rehabilitation

The foundation of successful cardiac rehabilitation lies in conducting a thorough examination of patients, which involves a comprehensive assessment of several aspects, including medical, psychological, and social elements that impact an individual's cardiovascular well-being [37]. The significance of these assessments cannot be overemphasized, as they serve as the fundamental basis for developing individualized rehabilitation strategies. This section focuses on the need for complete patient assessments [38]. It emphasizes their crucial role in maximizing results in cardiac rehabilitation. Furthermore, we investigate the utilization of risk stratification methods to customize rehabilitation strategies based on each patient's specific needs and hazards.

Importance of Comprehensive Patient Assessments

The comprehensive evaluation of patients in cardiac rehabilitation plays a crucial role in delivering individualized therapy, acknowledging the intricate nature of heart disease and its wide-ranging consequences. These assessments play a crucial role for various reasons. Identifying medical needs for cardiac events and disorders encompasses a broad spectrum of diverse manifestations and varying degrees of severity [39]. Through medical evaluations, healthcare professionals can discover the precise cardiovascular diagnosis, evaluate the degree of cardiac impairment, and establish the necessity for medical interventions such as pharmaceutical treatments, angioplasty, or surgical procedures. The assessments are crucial in developing rehabilitation regimens that prioritize safety and efficacy while considering the patient's medical condition and any contraindications [40]. The psychological well-being of individuals is frequently affected by cardiovascular disorders, resulting in the manifestation of symptoms such as anxiety, depression, and stress. Psychological assessments are crucial in identifying emotional issues impacting patient compliance with rehabilitation programs and overall recovery. The identification and mitigation of psychological distress play a pivotal role in promoting mental well-being and improving the patient's overall quality of life [35].

The influence of social support and lifestyle factors on cardiovascular health is substantial, as social determinants of health and various lifestyle choices significantly impact this aspect of well-being. Social assessments examine the patient's social support network, familial interactions, socioeconomic standing, and healthcare accessibility. Several factors can impact patients' capacity to alter their lifestyles and adhere to rehabilitation programs [21]. Healthcare providers can provide customized support and resources by gaining awareness of the social environment of their patients. Identifying risk factors is essential to patient assessments, encompassing a thorough examination of cardiovascular risk factors, including hypertension, hyperlipidemia, smoking, diabetes, and obesity [22]. Identifying and mitigating these risk variables is of utmost importance for secondary prevention, as unmanaged risk factors can significantly elevate the probability of recurring cardiovascular incidents. Healthcare practitioners utilize this data to formulate specific therapies to manage and mitigate these risks [23].

The evaluation of exercise tolerance is crucial to the customization of exercise recommendations. Cardiopulmonary and stress tests yield significant information regarding the individual's exercise capability and cardiovascular reaction to physical exertion [24]. These assessments play a crucial role in informing the formulation of exercise routines that are both safe and effective, thereby aiding patients in their journey to restore physical fitness and enhance their self-assurance. Baseline data is crucial for monitoring purposes in healthcare settings. Comprehensive evaluations play a vital role in establishing this baseline, enabling healthcare professionals to track and measure patient progress throughout rehabilitation [25]. The implementation of periodic evaluations enables healthcare professionals to effectively track alterations in medical conditions, psychological welfare, and the management of risk factors. The continuous feedback provided informs modifications to rehabilitation plans and guarantees that the changing demands of the patient are addressed [26].

Use of Risk Stratification Tools

Risk stratification techniques are crucial in customizing rehabilitation strategies according to individual patients' demands and hazards [31]. These methods facilitate classifying patients into risk groups by considering their clinical characteristics and prognosis, enabling healthcare providers to deploy resources and therapies efficiently [32]. Several risk stratification measures are frequently utilized in cardiac rehabilitation, including the Framingham Risk Score, the Duke Treadmill Score, and the Seattle Heart Failure Model.

The Framingham Risk Score is a predictive technique to assess the likelihood of experiencing coronary heart disease events over ten years. It considers many characteristics like age, gender, total cholesterol levels, high-density lipoprotein (HDL) cholesterol levels, blood pressure, diabetes status, and smoking history. Patients are categorized into low, middle, or high-risk groups [31-35]. Using the Framingham Risk Score aids healthcare professionals in customizing interventions to mitigate cardiovascular risk, encompassing lifestyle adjustments and pharmacological interventions. The Duke Treadmill Score is predominantly utilized in patients suspected of having coronary artery disease. This tool integrates the results of an exercise test, such as the duration of exercise and the presence of ST-segment depression, along with pertinent clinical information, including age, gender, and history of myocardial infarction. The purpose of this score is to forecast the probability of future cardiac events [34]. The Duke Treadmill Score guides decisions on subsequent diagnostic evaluations, therapies, and exercise recommendations [35].

The Seattle Heart Failure Model is a risk stratification tool designed specifically for individuals with heart failure. It utilizes clinical factors, laboratory data, and pharmaceutical information to forecast the likelihood of survival for these patients. It facilitates the customization of management strategies, encompassing modifications in medication, recommendations for implantable devices, and implementing innovative therapies for heart failure [36]. Risk stratification tools play a crucial role in optimizing the accuracy of rehabilitation plans by selecting suitable therapies and determining the level of monitoring required. For instance, people classified as high-risk may necessitate more regular evaluations, specific therapies, and closer monitoring [37]. In contrast, low-risk individuals may experience advantages from less rigorous surveillance. Furthermore, these instruments also aid in establishing attainable objectives for patients, guaranteeing that rehabilitation plans are secure and efficacious [38].

In summary, it can be stated that thorough patient evaluations are essential components of successful cardiac rehabilitation programs, as they establish the fundamental basis for individualized treatment. The assessments comprehensively evaluate physical, psychological, and social issues, acknowledging the intricate and interconnected aspects of cardiovascular health [39]. These tools empower healthcare providers to customize rehabilitation plans according to each patient's unique needs, effectively handle risk factors, and maximize desired outcomes. In addition, risk stratification techniques improve the accuracy of these evaluations, facilitating the distribution of resources and therapies according to patients' attributes and projected outcomes [40]. By integrating comprehensive evaluations and risk stratification, cardiac rehabilitation can be transformed into a personalized and evidence-based methodology, enabling individuals to regain control over their cardiovascular well-being and mitigate the likelihood of recurring episodes.

Exercise training in cardiac rehabilitation

Exercise training is vital in cardiac rehabilitation, providing a comprehensive strategy for enhancing cardiovascular well-being. The intervention above has a crucial and central role in optimizing recovery, mitigating recurrence risks, and improving the general well-being of patients afflicted with cardiovascular illnesses [11]. This section focuses on the current guidelines for exercise prescription in cardiac rehabilitation, examining the advantages of aerobic and resistance training in promoting cardiovascular well-being [12].

Latest Guidelines for Exercise Prescription

Cardiac rehabilitation programs conform to evidence-based recommendations for exercise prescription, which have undergone modifications throughout the years to incorporate advancements in research and clinical practice. The primary objective of these guidelines is to guarantee the safety, efficacy, and customization of exercise training following the specific requirements of each patient [13]. According to the most recent update in September 2021, the American Heart Association (AHA), the American College of Cardiology (ACC), and the American Association of Cardiovascular and Pulmonary Rehabilitation (AACVPR) have issued comprehensive guidelines about exercise training in the context of cardiac rehabilitation [14].

Aerobic exercise plays a vital role in cardiac rehabilitation by enhancing cardiovascular fitness and endurance and optimizing oxygen utilization by the heart and muscles. According to the guidelines, it is recommended that cardiac rehabilitation programs incorporate a minimum of 150 minutes of aerobic activity per week, characterized by a moderate-intensity level [15]. This exercise regimen should be spread out over three to five days. In an alternative approach, a recommended prescription for weekly aerobic exercise involves engaging in 75 minutes of vigorous-intensity activity. It is recommended that these exercise sessions be conducted under supervision and customized to accommodate the exercise capabilities of each patient [16]. The primary objective should be to incrementally enhance both the intensity and duration of the exercises as time progresses. Resistance training, often known as strength training, constitutes a crucial component of cardiac rehabilitation [17]. The activity entails engaging in physical workouts focusing on the major muscle groups by utilizing resistance, such as weights or resistance bands. The guidelines suggest including weight exercise in cardiac rehabilitation programs, with a minimum frequency of two sessions per week on days that are not consecutive. It is recommended that patients engage in one to three sets of eight to 12 repetitions for each exercise, gradually increasing the resistance over time to improve muscle strength and endurance [18].

Flexibility and balance exercises are advocated as supplementary components to aerobic and weight training to enhance range of motion and stability [19]. These exercises have the potential to aid people in restoring their functional capacity and mitigating the likelihood of falls, which is particularly pertinent for the elderly population [20]. The use of stretching exercises that specifically target the major muscle groups is recommended as part of the rehabilitation regimen. The process of prescribing exercise in cardiac rehabilitation necessitates tailoring the regimen to the unique characteristics of each patient, including their clinical condition, risk factors, comorbidities, and capacity for physical activity [21]. Risk stratification methods, such as the Duke Treadmill Score and the Seattle Heart Failure Model, can be utilized to classify patients into different risk groups, which can then inform the appropriate level of intensity and monitoring for exercise sessions. Individuals at higher risk may require increased monitoring and adjustments to their exercise routines to ensure their safety [22].

Benefits of Aerobic Exercise for Cardiovascular Health

Enhanced Cardiovascular Fitness: Aerobic exercise provides numerous advantages in terms of cardiovascular health. Consistent engagement in aerobic exercise has been shown to improve the heart's capacity to circulate blood, increasing stroke volume and cardiac output efficiently. Enhanced cardiovascular fitness reduces the burden placed on the heart, resulting in decreased resting heart rate and blood pressure [23]. The augmentation of endurance can be achieved through aerobic training, which enhances the muscles' capacity to utilize oxygen effectively. This implies that individuals can partake in their everyday routines with reduced exertion, encounter diminished levels of exhaustion, and engage in leisurely pursuits with enhanced convenience [24]. Weight control is facilitated by aerobic activity, as it aids in the expenditure of calories and reduces adipose tissue. Maintaining optimal body weight is paramount to mitigating the likelihood of developing cardiovascular ailments [25]. The blood lipid profile can be positively influenced by engaging in regular aerobic exercise, as it has the potential to elevate levels of high-density lipoprotein (HDL) cholesterol, commonly referred to as "good" cholesterol, while simultaneously decreasing levels of low-density lipoprotein (LDL) cholesterol. The observed improvement in the lipid profile is correlated with a decreased susceptibility to atherosclerosis [26].

Engaging in aerobic exercise positively influences the enhancement of insulin sensitivity and the regulation of blood glucose levels. This intervention holds particular advantages for people diagnosed with diabetes or those predisposed to the onset of this medical disease [27]. The regulation of blood pressure is a crucial aspect of maintaining cardiovascular health. Research has demonstrated that aerobic exercise can effectively reduce both systolic and diastolic blood pressure levels, hence significantly enhancing overall blood pressure management [28].

Benefits of Resistance Training for Cardiovascular Health

Resistance exercise provides distinct benefits for cardiovascular health. Resistance exercise has been shown to increase muscle strength and endurance, resulting in enhanced functional capacity [29]. Robust musculature can provide enhanced support and safeguard joints, mitigating the likelihood of sustaining injuries and experiencing falls. Enhancing metabolic health can be achieved through resistance training to develop lean muscle mass, elevating the resting metabolic rate [30]. This increase in metabolic rate can facilitate the process of fat loss and contribute to improved weight control. Insulin sensitivity and glucose uptake by muscles can be enhanced through resistance training, providing notable advantages for those with diabetes or those susceptible to developing the condition [31].

Cardiovascular Risk Factors: Existing research indicates that resistance training has the potential to reduce blood pressure and enhance lipid profiles, but to a lesser extent when compared to aerobic exercise [32]. Resistance training has positively impacted bone health by increasing bone density and reducing susceptibility to osteoporosis and fractures. These benefits are particularly significant for cardiovascular consequences, as they enhance mobility and general health [33].

In brief, exercise training is essential to cardiac rehabilitation, providing many advantages for cardiovascular well-being. Aerobic exercise has positively affected cardiovascular fitness, endurance, and metabolic health. On the other hand, resistance training has been shown to promote muscle strength, metabolic parameters, and bone health [34]. The most recent recommendations for exercise prescription emphasize the principles of individualization, risk stratification, and progressive development of exercise intensity and duration [35]. By integrating evidence-based exercise regimens into cardiac rehabilitation programs and customizing them to suit the unique requirements of each patient, healthcare providers enable individuals to maximize their cardiovascular health, mitigate the likelihood of recurring events, and improve their overall well-being [36].

Nutrition and lifestyle modification

Cardiac rehabilitation is a holistic strategy aimed at enhancing cardiovascular well-being, incorporating dietary guidelines and lifestyle modifications that can significantly influence overall results [36]. These therapies are designed to address risk factors and enhance the management of cardiovascular illnesses. This part focuses on discussing dietary advice, lifestyle adjustments, weight management, smoking cessation, and stress reduction, emphasizing their importance in cardiac rehabilitation [37].

Dietary Recommendations

Dietary recommendations are of paramount importance in maintaining cardiovascular health. Cardiac rehabilitation programs commonly prioritize the following dietary recommendations: Patients are advised to adhere to a heart-healthy dietary pattern that prioritizes the consumption of fruits, vegetables, whole grains, lean sources of protein, and low-fat dairy products [38]. The dietary pattern, often known as the Dietary Approaches to Stop Hypertension (DASH) diet, is characterized by its high nutrient content, specifically regarding heart health support. Including essential components such as fiber, potassium, and antioxidants achieves this. It is imperative to restrict the consumption of saturated and trans fats to regulate cholesterol levels effectively [39]. It is recommended that patients limit their intake of red meat, full-fat dairy products, and meals that contain partially hydrogenated oils.

The management of blood pressure is contingent upon a reduction in sodium consumption. It is recommended that patients restrict their intake of high-sodium foods, including processed and restaurant-prepared meals, and opt for using herbs and spices as flavor enhancers instead of salt. Monitoring portion sizes is crucial to managing caloric intake and sustaining a healthy body weight effectively [5]. Patients acquire the ability to identify suitable portion sizes and refrain from excessive consumption. The attainment of a harmonious distribution of macronutrients, namely carbs, proteins, and fats, holds paramount importance in promoting holistic well-being. Dietitians assist individuals in achieving optimal nutritional equilibrium by considering their unique requirements and dietary inclinations [6].

Lifestyle Changes

Weight management is of utmost importance for people with cardiovascular disorders, as it is essential to attain and sustain a healthy weight. When weight loss is deemed required, it can enhance blood pressure, cholesterol levels, and insulin sensitivity. Smoking cessation is a critical component in mitigating the risk of cardiovascular illnesses [7]. Cardiac rehabilitation programs offer a range of resources and support services to assist people in smoking cessation and mitigate the likelihood of heart-related issues [8]. Physical activity is a fundamental component of cardiac rehabilitation since it is crucial to enhancing cardiovascular fitness and maintaining general health and wellness. Healthcare providers recommend regular physical activity to patients as a means of encouragement. The detrimental impact of chronic stress on cardiovascular health is well documented [9]. Daily cardiac rehabilitation programs use stress management techniques, including mindfulness, relaxation exercises, and stress-reduction tactics.

Pharmacotherapy and Medical Management

Pharmacotherapy is crucial in managing cardiovascular illnesses and is frequently incorporated into cardiac rehabilitation programs. Antiplatelet agents, such as aspirin and clopidogrel, are often prescribed medications that mitigate the likelihood of blood clot formation and associated cardiovascular events [31]. Statins, also known as HMG-CoA reductase inhibitors, are pharmacological agents employed to reduce cholesterol levels and mitigate the likelihood of atherosclerosis and myocardial infarctions. Beta-blockers are a class of medications utilized for managing blood pressure, alleviating the heart's strain, and enhancing heart failure symptoms [31]. Angiotensin-converting enzyme inhibitors (ACE inhibitors) and angiotensin receptor blockers (ARBs) are pharmacological agents for managing hypertension and heart failure. Their mechanism of action involves the relaxation of blood vessels and alleviating cardiac burden [32]. Antiarrhythmic medications are commonly administered in the treatment of arrhythmias to manage and restore normal cardiac rhythm effectively.

The existing body of research provides substantial data supporting the effectiveness of these drugs in enhancing cardiovascular outcomes [3]. Multiple clinical trials and research have provided evidence of their efficacy in mitigating the likelihood of recurrent cardiovascular events and enhancing the overall prognosis [4]. Nevertheless, it is imperative to closely observe patients for potential adverse reactions and modify pharmaceutical regimens to achieve maximum therapeutic advantages.

Psychosocial Support

Psychosocial support refers to providing assistance and care that addresses individuals' psychological and social well-being. The psychosocial ramifications of heart disease for individuals and their families are considerable. A cardiac episode can elicit emotions such as dread, worry, depression, and uncertainty over one's future [5]. Cardiac rehabilitation programs acknowledge the need for psychosocial assistance and provide approaches to tackle mental health issues. Psychological assessment is a commonly employed method for identifying and treating emotional concerns in patients. Psychologists and counselors offer assistance and techniques for managing and adapting to challenging situations [6]. Education is provided to patients and their families regarding the emotional elements of heart disease, aiming to normalize their feelings and equip them with strategies to manage stress and anxiety effectively [7]. Support groups provide patients with a valuable platform to establish connections with individuals confronting comparable difficulties, cultivating a sense of community and empathy. Including family members in the rehabilitation process has been found to offer significant emotional support and enhance compliance with lifestyle modifications and medication regimens [8]. The integration of stress-reduction approaches, including relaxation exercises, mindfulness, and cognitive-behavioral therapy, is observed within cardiac rehabilitation programs to manage stress [9].

In summary, integrating diet and lifestyle modification with pharmaceuticals serves as the fundamental basis for cardiac rehabilitation [9]. The primary objective is to increase cardiovascular outcomes and promote the overall welfare of patients afflicted with heart disease. The primary emphasis of dietary recommendations is on adopting eating patterns that promote cardiovascular health. In addition, lifestyle modifications involve several aspects, such as weight management, smoking cessation, engagement in physical activity, and reducing stress levels. The utilization of pharmacotherapy is of paramount importance in the field of medical treatment since it is supported by robust empirical evidence that confirms its effectiveness [1-4]. Furthermore, it is imperative to acknowledge the significance of psychosocial support in addressing the psychological ramifications of heart disease, as it contributes to the comprehensive treatment of patients and their families. This recognition stems from the understanding that emotional well-being is crucial to maintaining cardiovascular health [8]. Cardiac rehabilitation programs are strategically developed to offer inclusive assistance, enabling individuals to implement enduring modifications that mitigate the likelihood of recurring cardiovascular incidents and enhance their overall well-being [9].

Quality of life and functional outcomes

The implementation of cardiac rehabilitation programs significantly influences the quality of life, functional capacity, and general well-being of individuals recovering from cardiovascular events or actively managing chronic heart problems [10]. The existing body of research and patient testimonies provide substantial evidence supporting the beneficial impact of cardiac rehabilitation on these particular features [10].

Impact on Quality of Life

Multiple studies have provided evidence that participation in cardiac rehabilitation programs leads to notable enhancements in participants' quality of life. Quality of life involves various aspects, including physical, psychological, and social well-being. Cardiac rehabilitation programs aim to address each of these dimensions [11]. Physical well-being encompasses various aspects of an individual's health, including energy levels, fatigue symptoms, and daily activity performance [12]. It has been observed that patients frequently report enhancements in these areas, such as heightened energy levels, diminished tiredness symptoms, and enhanced capacity to engage in routine tasks. Individuals feel a restoration of self-assurance in their physical aptitudes and encounter a reduction in the constraints they face in their everyday activities [13]. Cardiac rehabilitation programs include systematic exercise regimens that effectively enhance cardiovascular fitness, facilitating the resumption of patients' customary pursuits encompassing both occupational and recreational endeavors [14].

Psychological well-being is a crucial aspect of cardiac rehabilitation programs since they integrate psychological support services to manage the emotional impact associated with cardiovascular disorders effectively. Patients are provided counseling on effectively managing stress, anxiety, and depression, all frequently experienced following cardiac incidents [2]. Psychological therapies, such as cognitive-behavioral therapy and mindfulness techniques, provide patients with effective coping mechanisms to manage the emotional issues commonly associated with cardiac disease. Consequently, the participants reported a decrease in anxiety and depression symptoms and a general enhancement in their emotional well-being [7]. The promotion of social well-being is facilitated by the establishment of a supportive community among patients participating in cardiac rehabilitation programs, which is characterized by a sense of camaraderie and the sharing of experiences [8]. The inclusion of support groups in these programs facilitates a forum for individuals to establish connections, exchange personal narratives, and give reciprocal motivation. The presence of this social support network can exert a significant favorable influence on patients' emotional resilience and overall sense of belonging [9].

Functional Capacity and Independence

Cardiac rehabilitation prioritizes the enhancement of functional capacity. This is especially critical for people recuperating after cardiac events or surgical procedures, as they frequently encounter a deterioration in their physical capabilities. Implementing systematic exercise training within cardiac rehabilitation programs facilitates the restoration of patients' physical strength, endurance, and mobility, enabling them to resume their routine activities effectively [9]. Case studies and patient testimonials provide further evidence to demonstrate the significant effects of cardiac rehabilitation on functional results. As an illustration, a patient who had undergone coronary artery bypass surgery and afterward engaged in a cardiac rehabilitation program recounted their triumph [10]. Before undergoing rehabilitation, individuals experienced difficulty breathing, even when engaging in ordinary daily tasks. By following the program's workout regimen and receiving guidance on lifestyle improvements, individuals experienced enhancements in their cardiovascular fitness. They restored their capacity to partake in extended walks and leisure activities alongside their loved ones. This enhanced their physical well-being, general life satisfaction, and perception of autonomy [12]. Additionally, it is worth noting that cardiac rehabilitation has a range of beneficial impacts that go beyond the realm of physical well-being. Frequently, patients express a revitalized sense of purpose and a dedication to embracing practices that promote cardiovascular health [13]. Individuals assume the role of health advocates, assuming responsibility for their well-being through diligent adherence to prescribed drug regimens, adoption of dietary enhancements, and consistent engagement in exercise programs [14].

Challenges and barriers to accessing cardiac rehabilitation

Despite the many advantages associated with cardiac rehabilitation, some significant obstacles and impediments impede individuals' ability to access and complete these programs. One notable obstacle that hinders progress is the comparatively low rate at which healthcare providers make referrals. There exists a subset of patients who may benefit from engaging in cardiac rehabilitation [15]. Yet, it has been observed that a proportion of these individuals are not being referred for such programs. This occurrence can be attributed to insufficient knowledge among healthcare practitioners on the benefits of cardiac rehabilitation and the perception that specific patients may not be good candidates for such interventions [16]. Underutilization of cardiac rehabilitation programs is observed since a discrepancy exists between the number of eligible patients referred and the enrollment rate. The barriers encompass transportation challenges, insufficient insurance coverage, inconvenient program hours, and conflicts arising from work commitments [7].

Cultural and socioeconomic characteristics have been identified as influential determinants of participation in cardiac rehabilitation, with socioeconomic status being particularly significant. This encompasses various dimensions, such as income, education, and healthcare access, collectively shaping individuals' engagement with cardiac rehabilitation programs [16]. Engagement may be influenced by cultural attitudes and linguistic difficulties, particularly within diverse communities [17]. The geographical proximity to cardiac rehabilitation centers might deter participation, particularly in rural or impoverished regions characterized by restricted access to healthcare resources. Both patients and healthcare providers may possess little knowledge regarding the advantages of cardiac rehabilitation and the accessibility of nearby programs [18].

Strategies for Overcoming Barriers

The endeavor to surmount these hurdles and barriers holds paramount importance in guaranteeing that individuals attain the complete advantages of cardiac rehabilitation. Cardiac rehabilitation's significance necessitates enhancing awareness among healthcare practitioners, patients, and the community [19]. Educational campaigns have the potential to highlight their favorable influence on health outcomes and overall quality of life. The provision of patient navigation services has been shown to assist individuals in overcoming logistical difficulties, including those related to transportation and insurance [30]. Navigators can guide patients during the enrolling process and extend support throughout their rehabilitation trajectory.

Tailored programs provide a flexible approach to assist patients who face constraints related to their work schedules or transportation options [21]. The emergence of telerehabilitation options has shown promise as a viable approach for improving access to rehabilitation services, enabling patients to participate in therapy remotely [22].

Culturally Competent Care: Implementing culturally adapted interventions and materials can effectively overcome language obstacles and cultural preferences, enhancing the inclusivity and efficacy of cardiac rehabilitation. Promoting physician engagement is crucial to fostering proactive discussions and cardiac rehabilitation recommendations among eligible healthcare patients [23]. Implementing integrated care models encompassing primary care doctors, cardiologists, and rehabilitation specialists can potentially optimize the process of referrals and enhance patient participation. In summary, it can be observed that cardiac rehabilitation significantly affects the quality of life, functional outcomes, and overall well-being of persons diagnosed with cardiovascular disorders [24]. The research is substantiated by patient testimonials and case studies that exemplify the transforming impacts of these programs [25]. Nevertheless, the accessibility and successful completion of cardiac rehabilitation might be hindered by various obstacles and limitations, such as inadequate referral rates, insufficient usage, and the influence of cultural and socioeconomic variables [26]. Implementing many techniques, including education, patient navigation, personalized programs, cultural competency, and physician engagement, plays a vital role in overcoming these barriers and ensuring that individuals receive comprehensive care that maximizes their cardiovascular health and overall well-being [27].

Cost-effectiveness and healthcare policy

Cardiac rehabilitation programs have been found to yield substantial advantages in boosting cardiovascular outcomes and augmenting the quality of life for patients [28]. Nevertheless, evaluating their cost-effectiveness is imperative, as cardiovascular illnesses' economic impact on healthcare systems is substantial. Furthermore, the impact of healthcare policy and reimbursement models is significant in shaping the availability and consumption of cardiac rehabilitation therapies [29].

Cost-effectiveness of Cardiac Rehabilitation

Multiple studies have examined the cost-effectiveness of cardiac rehabilitation programs, and the findings generally indicate that these programs are a prudent allocation of resources for healthcare systems [23]. Research studies have demonstrated that these programs can effectively diminish healthcare consumption and expenses by mitigating subsequent cardiovascular events, hospital admissions, and expensive medical interventions, such as revascularization procedures or heart transplants [24]. The long-term benefits of cardiac rehabilitation highlight the cost-effectiveness of this intervention. It has been observed that individuals who successfully undergo cardiac rehabilitation exhibit enhanced health outcomes, decreased death rates, and a reduction in disability-adjusted life years (DALYs) lost as a result of cardiovascular incidents. These characteristics result in significant cost reductions for healthcare systems and society [28].

Furthermore, it is essential to note that the advantages of cardiac rehabilitation go beyond just reductions in healthcare expenditures. Enhanced functional capability and quality of life can result in heightened production and a more favorable economic perspective for individuals, indirectly fostering societal well-being [31].

Healthcare Policies and Reimbursement Models

Healthcare regulations and reimbursement structures substantially impact the utilization of cardiac rehabilitation therapies. Policies exhibit variation across different countries, yet it is vital to analyze many recurring themes that are commonly observed [32]. The scope of insurance coverage for cardiac rehabilitation therapy exhibits significant variability. Cardiac rehabilitation coverage varies across different nations, with certain countries offering comprehensive financial support through either public or private insurance schemes. However, in other jurisdictions, patients may encounter the need to bear the costs associated with such rehabilitation services personally [34]. Enhancing insurance coverage for cardiac rehabilitation can enhance the accessibility and utilization of this vital healthcare service. Healthcare policies frequently govern the procedure of provider referral to cardiac rehabilitation programs. In specific healthcare systems, the referral rates are not optimal due to healthcare personnel lacking awareness or inadequate incentives for making referrals [34]. Implementing policies that facilitate automated referrals or offer incentives to clinicians can effectively mitigate this issue [35].

The accreditation of cardiac rehabilitation programs might exhibit variability in criteria, influencing the quality and uniformity of care provided. Implementing well-defined and stringent accreditation requirements, along with the provision of financial incentives, can motivate programs to uphold elevated levels of care [3]. The advancements in telehealth and remote monitoring have emerged as significant innovations with the potential to enhance the accessibility of cardiac rehabilitation therapies, particularly in geographically isolated or underserved regions [33]. Policymakers must deliberate upon regulatory frameworks and reimbursement models that facilitate the integration of telemedicine into cardiac rehabilitation programs [34]. Value-based payment models, which connect remuneration and patient outcomes, can motivate healthcare providers to provide superior-quality cardiac rehabilitation services and prioritize patient-centered care.

In brief, the accessibility and utilization of cardiac rehabilitation therapies are significantly influenced by healthcare policy and reimbursement models. Implementing policies to facilitate insurance coverage, provider referral, program accreditation, telehealth adoption, and value-based payment models can potentially improve access to and the overall efficacy of cardiac rehabilitation programs [31-35].

Potential areas for future research

The ongoing development of cardiac rehabilitation warrants consideration of several developing trends and prospective directions. The emergence of precision medicine and genomics has the potential to significantly transform the field of cardiac rehabilitation through the facilitation of individualized treatment strategies [35]. Using genetic and molecular profiling techniques enables the identification of individuals with a heightened genetic predisposition to cardiovascular illnesses. This information may then be utilized to customize interventions specifically tailored to the particular genetic composition of these individuals [36]. Virtual reality (VR) and gamification are now being investigated as potential strategies to augment engagement levels in the context of cardiac rehabilitation. The utilization of these technologies has the potential to enhance the enjoyment and immersion of workout routines, hence potentially enhancing adherence to rehabilitation programs [37].

The significance of integrated digital health platforms in cardiac rehabilitation is growing steadily. These platforms can provide remote monitoring, offer instructional resources, and permit real-time contact between patients and healthcare practitioners. The popularity of home-based cardiac rehabilitation programs is increasing as they offer patients the opportunity to engage in rehabilitation activities inside the confines of their residences [38]. This particular methodology has the potential to improve accessibility, particularly for people who face challenges related to transportation or limited mobility [39]. Behavioral therapies, such as cognitive-behavioral therapy and motivational interviewing, are being incorporated into cardiac rehabilitation programs to target psychological issues that impact cardiovascular health. These therapies can improve patients' emotional well-being and facilitate compliance with lifestyle adjustments [40].

Future cardiac rehabilitation programs may focus on targeting populations with a higher risk, including individuals with numerous comorbidities, those who have experienced recurrent cardiovascular events, and younger patients with a familial propensity to heart disorders [22]. The prioritization of expanding cardiac rehabilitation services to disadvantaged regions and emerging countries is of global significance. The growth process necessitates implementing crucial measures such as enhancing awareness, providing training to healthcare professionals, and tailoring programs to align with the specific cultural contexts of the local communities [24]. Continuous research plays a crucial role in enhancing the body of data supporting cardiac rehabilitation. Examining novel therapies, diverse patient groups, and long-term results has the potential to enhance the precision of medical practice and the quality of patient care [25].

In summary, the potential of cardiac rehabilitation in the future is promising, as it is propelled by advancements in precision medicine, digital health technologies, and a focus on patient-centered treatment. The aforementioned new developments hold the potential to augment the accessibility, efficacy, and individualized characteristics of cardiac rehabilitation programs [26]. In addition, the potential influence of precision medicine and genomics on individualized treatment strategies presents novel opportunities for enhancing cardiovascular outcomes and enhancing the general welfare of persons afflicted with cardiovascular conditions [27]. In light of the ongoing evolution within the discipline, healthcare systems and policymakers must remain cognizant of these advancements and modify policies and practices accordingly to facilitate the changing landscape of cardiac rehabilitation [30].

## Conclusions

In conclusion, this extensive literature review has provided an in-depth discussion of the complex field of cardiac rehabilitation in contemporary times. It has covered various aspects, including its historical development, epidemiological importance, diverse stages, patient evaluations, physical exercise regimens, dietary and lifestyle adjustments, medication interventions, psychological assistance, and economic viability. The unequivocal beneficial effects of cardiac rehabilitation on patient outcomes have been thoroughly examined, encompassing several aspects such as heightened quality of life, greater functional capacity, and decreased mortality rates. However, it is essential to acknowledge that some ongoing challenges and obstacles persist, including underutilization and socioeconomic inequities. These hurdles must be effectively addressed and overcome to make meaningful progress. In anticipation of forthcoming developments, the future of cardiac rehabilitation exhibits potential in tailored treatment strategies propelled by advancements in precision medicine and technological breakthroughs. Given the ongoing evolution of this field, it is becoming increasingly crucial for healthcare systems and politicians to adjust and guarantee fair access to these essential programs. This adaptation is essential to effectively mitigating the impact of cardiovascular disease on both people and society as a whole.
